# SARS-CoV-2 Shedding from Asymptomatic Patients: Contribution of Potential Extrapulmonary Tissue Reservoirs

**DOI:** 10.4269/ajtmh.20-0279

**Published:** 2020-05-13

**Authors:** Raj Kalkeri, Scott Goebel, Guru Dutt Sharma

**Affiliations:** 1 Southern Research, Frederick, Maryland;; 2 College of Optometry, Western University of Health Sciences, Pomona, California

## Abstract

The ongoing pandemic COVID-19, caused by SARS-CoV-2, has already resulted in more than 3 million cases and more than 200,000 deaths globally. Significant clinical presentations of COVID-19 include respiratory symptoms and pneumonia. In a minority of patients, extrapulmonary organs (central nervous system, eyes, heart, and gut) are affected, with detection of viral RNA in bodily secretions (stool, tears, and saliva). Infection of such extrapulmonary organs may serve as a reservoir for SARS-CoV-2, representing a potential source of viral shedding after the cessation of respiratory symptoms in recovered patients or in asymptomatic individuals. It is extremely important to understand this phenomenon, as individuals with intermittent virus shedding could be falsely identified as reinfected and may benefit from ongoing antiviral treatment. The potential of SARS-CoV-2 infection to rapidly disseminate and infect extrapulmonary organs is likely mediated through the nonstructural and accessory proteins of SARS-CoV-2, which act as ligands for host cells, and through evasion of host immune responses. The focus of this perspective is the extrapulmonary tissues affected by SARS-CoV-2 and the potential implications of their involvement for disease pathogenesis and the development of medical countermeasures.

## INTRODUCTION

The current pandemic COVID-19 caused by SARS-CoV-2 is rapidly spreading across the globe, with more than 3 million infections and more than 200,000 deaths worldwide. The receptor of SARS-CoV-2, angiotensin converting enzyme 2 (ACE2), is expressed in the lungs, heart, kidneys, intestines, brain, eyes, and testicles.^1,2^ Infection of these extrapulmonary organs (eyes, gastrointestinal tract, and brain)^3^ has been reported. Viral shedding in asymptomatic individuals and recovered patients after the cessation of respiratory symptoms^4,5^ has been documented. Although SARS-CoV-2 positivity of recovered patients may be interpreted as reinfection, failure to reinfect monkeys in the laboratory setting^6^ argues against the possibility of reinfection and suggests the likelihood of extrapulmonary reservoirs in the infected individuals. Considering this possibility, this perspective is focused on extrapulmonary organs affected by SARS-CoV-2 and the implications of their involvement for disease transmission, clinical management strategies, and medical countermeasure discovery and development.

### SARS-CoV-2 and extrapulmonary tissues and organs.

In addition to the primary respiratory route of infection via droplets or contact with fomites, the expression of ACE2 in aqueous humor^7^ and neural tissue of the retina^8^ suggest a potential role of transmission via an ocular route. The ocular reservoir can harbor low viral load, even before transmission to other organs such as the throat or lungs, as 75% of tears drain into the inferior meatus of the nasal cavity and to the back of the throat.^9^ Red eyes, conjunctivitis, conjunctival hyperemia, chemosis, epiphora, or increased secretions are observed in a minority of patients, along with detectable SARS-CoV-2 RNA in tears.^10,11^ Although viral RNA is infrequently detected (1–5%) in tears, ocular manifestations are relatively common in COVID-19–positive patients (10–30%). This could be due in part to timing of sample collection, fluctuations in virus shedding, and variability in testing methods. Standardized approaches for sample collection along with more sensitive testing methods may yield more robust data. Additional research is needed to confirm the temporal correlation between conjunctivitis and viral shedding in COVID-19 patients.

The gastrointestinal tract is also affected by SARS-CoV-2. Diarrhea and shedding of SARS-CoV-2 in stool are reported in the literature.^12,13^ Currently, transmission through the fecal–oral route is not documented. However, it remains a possibility considering the detection of SARS-CoV-2 RNA in wastewater and municipal sewage.^14^ Fecal shedding also increases the risk of creating a new intermittent animal reservoir and emergence of new viral strains through recombination, which could serve as starting points of new outbreaks.

Neurological manifestations (headache, loss of taste and smell, dizziness, impaired consciousness, and epilepsy) are reported in some COVID-19 patients.^15^ SARS-CoV-2 RNA was also detected in the cerebrospinal fluid of a patient diagnosed with COVID-19 and viral encephalitis.^16^ It is postulated that coronaviruses can enter the central nervous system (CNS) via olfactory nerve, blood circulation, and neuronal pathways, leading to neurological abnormalities and symptoms.^17^ Liver, kidney, and heart abnormalities are also observed in COVID-19 patients,^18,19^ and although SARS-CoV-2 RNA is not reported in these tissues after autopsy, the detection of viral RNA in the liver of the hamster model^20^ suggests the infection of these organs in patients.

Although SARS-CoV-2 RNA is detected in the blood (1% of patients),^3^ at present, it is unknown if the virus is shed in breast milk, semen, or vaginal fluid. Extrapulmonary complications in COVID-19 patients include diarrhea (gastrointestinal tract), confusion (CNS), hepatic, and renal injury.^21^ Some of these complications may also be due to compromised pulmonary function. Extrapulmonary tissues affected by SARS-CoV-2 are listed in Table 1. Currently, it is unknown if SARS-CoV-2 can replicate in non-respiratory tissues (eyes, liver, and CNS) to produce infectious virus. However, SARS virus has been shown to replicate in human kidney (HEK293) and hepatic (Huh7 and HepG2)^22^ cell lines and detected in the liver and brain of patients.^23,24^ Experimental infection of primary tissue cells with SARS-CoV-2 and longitudinal studies in infected patients and animal models can promote a greater understanding of the role of these tissues in the infection.

**Table 1:** Extrapulmonary tissues affected by SARS-CoV-2

Organ	Clinical finding
Lungs^38^	Acute respiratory distress syndrome
Eyes^39^	Conjunctivitis
Liver^40,41^	Liver injury
Systemic circulation (blood)^3^	Thrombosis
Kidney^19,18^	Renal injury
Brain/CNS^1,42^	CNS symptoms
GI tract^13,12^	Diarrhea

CNS = central nervous system; GI = gastrointestinal.

Ocular and CNS tissues are considered immune-privileged sites.^25,26^ For other pathogens such as *Cytomegalovirus* (CMV), Zika virus, Ebola virus, and other beta coronaviruses (Table 2), these organs have been shown to serve as reservoirs, facilitating viral persistence.^27^ Many COVID-19 patients test positive even after discharge from the hospital.^28,29^ In one report, SARS-CoV-2 RNA was detected up to 60 days after the onset of symptoms and 36 days after complete resolution of symptoms in the patient’s nasopharyngeal and/or oropharyngeal swabs.^30^ Another study reported undetectable viral load on days 21 and 22 after symptom onset in oropharyngeal saliva samples of a COVID-19 patient, followed by viral RNA detection on days 23 and 24, without any detectable virus for the next 5 days.^31^ Taken together, reports of prolonged incubation periods where virus is shed from asymptomatic infected persons^4^ or recovered patients several days after disease symptoms with an intermittent period of shedding,^31^ along with the detection of SARS-CoV-2 in the extrapulmonary tissues, strongly suggest the presence of extrapulmonary SARS-CoV-2 tissue reservoirs. These extrapulmonary virus tissue reservoirs in infected patients may also explain the highly variable incubation period associated with the onset of symptoms after an initial exposure as well as the duration of time for complete viral clearance.

**Table 2:** Extrapulmonary tissue reservoirs of other coronaviruses

Organ	Species	Coronaviruses
Brain	Mice	SARS-CoV^43^
	Mice	MERS-CoV^44^
	Human	HCoV-229E^45^
	Mice	HCoV-OC43^46^
Liver	Human	SARS-CoV^23^
	Mice	Mouse hepatitis Virus (MHV-A59)^47^
Kidneys	Human	Endemic Balkan nephropathy virus^48^
GI tract	Human	HCoV-HKU1^49^

MERS = Middle Eastern Respiratory Syndrome-Corona Virus; HCoV = human corona virus.

### Role of SARS-CoV-2 proteins in immune evasion.

Nonstructural proteins (NSP1, 3, and 16) and accessory proteins (ORF 3a, 6, and 9b) of SARS-CoV-2 are thought to play a role in the evasion of host immune responses (Table 3). A recent report also predicted a potential role of SARS-CoV-2 NSP5 and NSP13 interfering with the host immune response.^32^ Considering the substantial sequence similarity of more than 80% between SARS and SARS-CoV-2 proteins (Table 3), it is quite possible that SARS-CoV-2 can also escape the host immune response using similar mechanisms in non-respiratory tissues such as the liver and kidneys.

**Table 3:** SARS-CoV-2 proteins, homology to SARS, and proposed impact on host immunity

Protein (SARS-CoV-2)	Homology with SARS (%)	Mechanism of immune suppression in SARS
NSP1	91.1	Host RNA degradation and immune suppression^50,51^
NSP3	86.5	Papain-like protease, deubiquitination, and host IRF3 function inhibition^52,53^
NSP16	98.0	2′O Methyltransferase. Cap methylation is necessary to evade immune response^54^
ORF 3a	85.1	Downregulation of type 1 IFN receptor^55^
ORF 6	85.7	Inhibition of STAT1 function^56^
ORF 9b	84.7	Degradation of MAVS, TRAF3, and TRAF 6^57^

NSP = nonstructural protein; ORE =accessory protein.

### Implications of SARS-CoV-2 infection in extrapulmonary tissues.

The presence of extrapulmonary tissue reservoirs enhances the risk of organ malfunction, such as abnormal liver or kidney functions and impaired nervous system, leading to exacerbated disease complications and delayed recovery time in COVID-19 patients. Tissue reservoirs in immunocompromised patients are a major concern as the virus could spread to the respiratory system at an opportune time, exerting a more aggressive clinical course. Reports of continued or delayed virus shedding up to 36 days after cessation of symptoms^30,33^ suggest that longer term monitoring of recovered COVID-19 patients and improved virus containment strategies will be required to mitigate further community transmission. Currently, the amount of virus present in the extrapulmonary reservoirs relative to the amount of virus shed, such as in aerosol droplets, is unknown. As different viral loads have been observed in various bodily fluids (saliva, tears, feces, throat, or nasal discharge), longitudinal testing of “paired samples” collected from these different sites may be needed.

The proportion of asymptomatic carriers potentially shedding the virus from both pulmonary and extrapulmonary virus reservoirs is estimated to be between 17.9%^34^ and 30.8%,^35^ suggesting the importance of population-based screening using sensitive and robust assays. For other viral diseases such as measles and norovirus infection, viral transmission from asymptomatic carriers is well documented.^36,37^ Hence, global harmonization of the sensitivity and robustness of SARS-CoV-2 detection kits and screening of populations at risk might ensure identification of asymptomatic carriers of infection.

Potential antiviral drugs against SARS-CoV-2 may need to demonstrate bioavailability in extrapulmonary tissue reservoirs outside of the lungs, raising concerns of adverse events. Achieving efficacious levels of therapeutics in some of these tissues may be challenging because of the presence of blood–brain and blood–retina barriers. Vaccine and antiviral candidates may also need to demonstrate efficacy in the prevention of tissue reservoirs, which could introduce additional stringency requirements for clinical trials.

Development of appropriate animal models can address some of these questions. Golden Syrian hamsters infected with SARS-CoV-2 exhibited contact transmission, weight loss, lung damage, intestinal mucosal inflammation, lymphoid atrophy, myocardial degenerative changes, and expression of viral nucleocapsid in lungs and intestines.^20^ Interestingly, viral RNA could be detected in extrapulmonary tissues such as the liver, heart, spleen, kidneys, brain, and salivary glands, confirming the extrapulmonary manifestation of SARS-CoV-2 disease. Although hamsters could be a cost-effective animal model for SARS-CoV-2, lack of hamster-specific immunological reagents and unknown utility for testing medical countermeasures could limit their role in SARS-CoV-2 preclinical studies. Rhesus monkeys have been successfully infected with SARS-CoV-2.^6^ Viral replication was observed in extrapulmonary tissues (gut, spinal cord, heart, skeletal muscles, and bladder). Reexposure of previously infected monkeys elicited no signs of viral replication in extrapulmonary tissues, suggesting it could be a useful animal model to study SARS-CoV-2 tissue reservoirs and efficacy of vaccines. However, it is also important to note the importance of inoculation dose, age of animals, and route of challenge (ocular, intranasal, or oral) in the development and utility of animal models to address different research questions.

Several scientific questions remain to be addressed to fully understand COVID-19 clinical disease progression, including potential differences in extrapulmonary tissue infections with respect to age or ethnicity. It will also be necessary to consider the kinetics and duration of viral shedding, which could be impacted by viral bio-distribution within and among different tissue reservoirs. In addition, the role of host immune responses and the expression of host factors must be considered as dynamic forces in driving genotypic or virologic differences among viral quasi-species isolated from different reservoirs. The identification of non-respiratory tissue reservoirs of SARS-CoV-2 suggests that further studies are needed to address implications for COVID-19 disease progression, effects on extrapulmonary tissues harboring the virus, and development of optimal medical countermeasures and disease management strategies.
